# Clinical Outcome of Indirect Bonded Porcelain Restoration Versus Full-Coverage Crown on Endodontically Treated Teeth in Posterior Areas: A Systematic Review

**DOI:** 10.7759/cureus.70116

**Published:** 2024-09-24

**Authors:** Mai M Alhamdan, Norah Alghuwainem, Mona Alharbi, Shoag Hummady

**Affiliations:** 1 Department of Prosthetic Dental Sciences, King Saud University, Riyadh, SAU; 2 Department of Dentistry, King Saud University, Riyadh, SAU

**Keywords:** ceramic crown, full converge crown, inlay and onlay, partial indirect bonded porcelain restoration, partial indirect bonded restoration

## Abstract

The significant loss of tooth structure that occurs after dental caries and endodontic therapy is a common clinical challenge. Several methods were discussed in the literature to treat badly damaged teeth. This systematic review aims to compare the clinical outcomes of partial indirect bonded porcelain restorations to full-coverage crowns of endodontically treated teeth in posterior areas. The study followed the Preferred Reporting Items for Systematic Reviews and Meta-Analysis (PRISMA) guidelines. Data included in the review were identified through bibliographic research on electronic databases (PubMed/Medline, Google Scholar, Cochrane Library, and Web of Science). The strategy was applied by searching for randomized clinical trials (RCTs), cohort studies, and case-control studies using keywords (partial indirect bonded porcelain restoration, partial indirect bonded restoration, inlay and onlay, full convergent crown, and ceramic crown). As a result of a literature search through selected databases using the keywords, 88,421 papers were identified. A total of 671 articles were related and screened for inclusion and exclusion criteria. Four hundred and eighty-two articles that did not meet the inclusion criteria were excluded. A total of 189 full-text papers were assessed, and 20 articles were included in this study. Nineteen cohort studies and one case-control study were included and assessed in this review. This study concludes that both full-coverage crowns and partial indirect bonded restorations demonstrate comparable survival rates. However, limited studies exist regarding partial indirect bonded restorations on endodontically treated teeth, indicating the need for further updated studies.

## Introduction and background

The considerable loss of tooth structure caused by dental caries and endodontic therapy is a typical clinical problem in daily dental practice [1]. This compromises the tooth's integrity and increases the probability of fracture [1]. The literature discusses several strategies for treating severely damaged teeth [2]. 

Currently, there are three treatment options for single endodontically treated teeth: direct adhesive restorations, indirect bonded restorations (BPRs), and conventional full-coverage crowns. The amount of residual sound tooth structure is the most critical factor that influences the treatment methods [2]. The greater the remaining tooth structure the greater the fracture resistance and the better the prognosis [2]. 

A full-coverage crown is an extra-coronal restoration that covers the outer surface of the clinical crown. It is widely used to restore severely damaged teeth both esthetically and functionally [1]. Some authors suggest inserting a post to reinforce the root followed by a crown; on the other hand, others suggest that intracanal retention weakens the tooth structure [1]. However, a full-coverage crown has been associated with secondary caries and underlying tooth fractures [3]. 

Furthermore, in recent years, the development of novel adhesive systems has led to a transformation in dental practice, driven by higher esthetic expectations and the growing demand for conservative treatments aligned with the principles of minimally invasive dentistry [4]. As a result, indirect BPR was introduced to dentistry. 

Indirect BPRs represent a conservative approach that offers both esthetic and functional benefits, with success largely depending on the integrity of the remaining tooth structure [4]. The use of partial adhesive restorations on vital and endodontically treated teeth revealed variable long-term clinical outcomes, indicating that endodontically treated teeth are more prone to failure than vital teeth [5]. 

The success of the restoration is determined by a variety of criteria, including material selection, restoration design, occlusion, and cementation media [6]. One study indicated that long-term success is influenced by the dentist's operating decisions based on an individual clinical case: direct or indirect restorations, overlays or full crowns, post-placement, material quality, and preparation design principles [2]. 

Many clinical studies have been conducted to evaluate the clinical consequences of both full-coverage crowns and partial BPR in endodontically treated teeth. However, according to the researchers' findings, no systematic review has yet been done to compare full-coverage crowns versus partial BPR restorations.

The aim of this systematic review is to compare the clinical outcomes of partial indirect BPRs versus full-coverage crowns for teeth that were endodontically treated in posterior regions.

Research question (PICO) 

To compare the clinical outcomes and survival rates of endodontically treated teeth in posterior areas treated with full-coverage crowns or indirect partial BPR. 

## Review

Methodology

Studies Eligibility Criteria

Inclusion criteria: This systematic review will include studies conducted in the past 20 years in English of endodontically treated teeth, placed in posterior areas, and restored with full-coverage crowns or partial BPR. Research designs will include randomized clinical trials (RCTs), cohort studies, and case-control studies. 

Exclusion criteria: Any study conducted in a language other than English, older than 20 years ago, or using restoration materials other than ceramic or porcelain materials, or any research design other than RCTs, cohort studies, and case-control studies will be excluded.

Data Sources and Search Strategy

The study will follow the Preferred Reporting Items for Systematic Reviews and Meta-Analysis (PRISMA) guidelines [7]. Data that will be included in the review will be identified through bibliographic research on electronic databases (PubMed/Medline, Google Scholar, Cochrane Library, and Web of Science). The strategy will be applied by searching for RCTs, cohort studies, and case-control studies by four data collectors and reviewers working independently. Endnote will be used for citation and data duplication removal.

Keywords used:* *Partial indirect bonded porcelain restoration, partial indirect bonded restoration, inlay and onlay, full convergent crown, ceramic crown.

Data Extraction 

All electronic databases mentioned will be screened by the three reviewers to identify the included titles. The articles that meet the inclusion criteria will be labeled by the name of the database, the title, and the author’s name. The data extracted will include longevity, type of treatment, follow-up period, and failure. 

Quality Assessment of Selected Articles and Bias Evaluation 

After data extraction, the final studies included in the review will be assessed by the reviewers using different scales to evaluate the risk of bias. According to Susan Armijo Olivo [8], the Jadad Scale (JS) presented the best validity and reliability evidence to test the quality of RCTs [9], and this scale will be applied in this study. The Newcastle-Ottawa Scale (NOS) will be used for the evaluation of cohort and case-control studies [10].

*Ethical Consideration* 

Ethical approval for this research was obtained from the College of Dentistry Research Center, CDRC, King Saud University, with Reg. No. IR 0474. 

Results 

**Figure 1:**
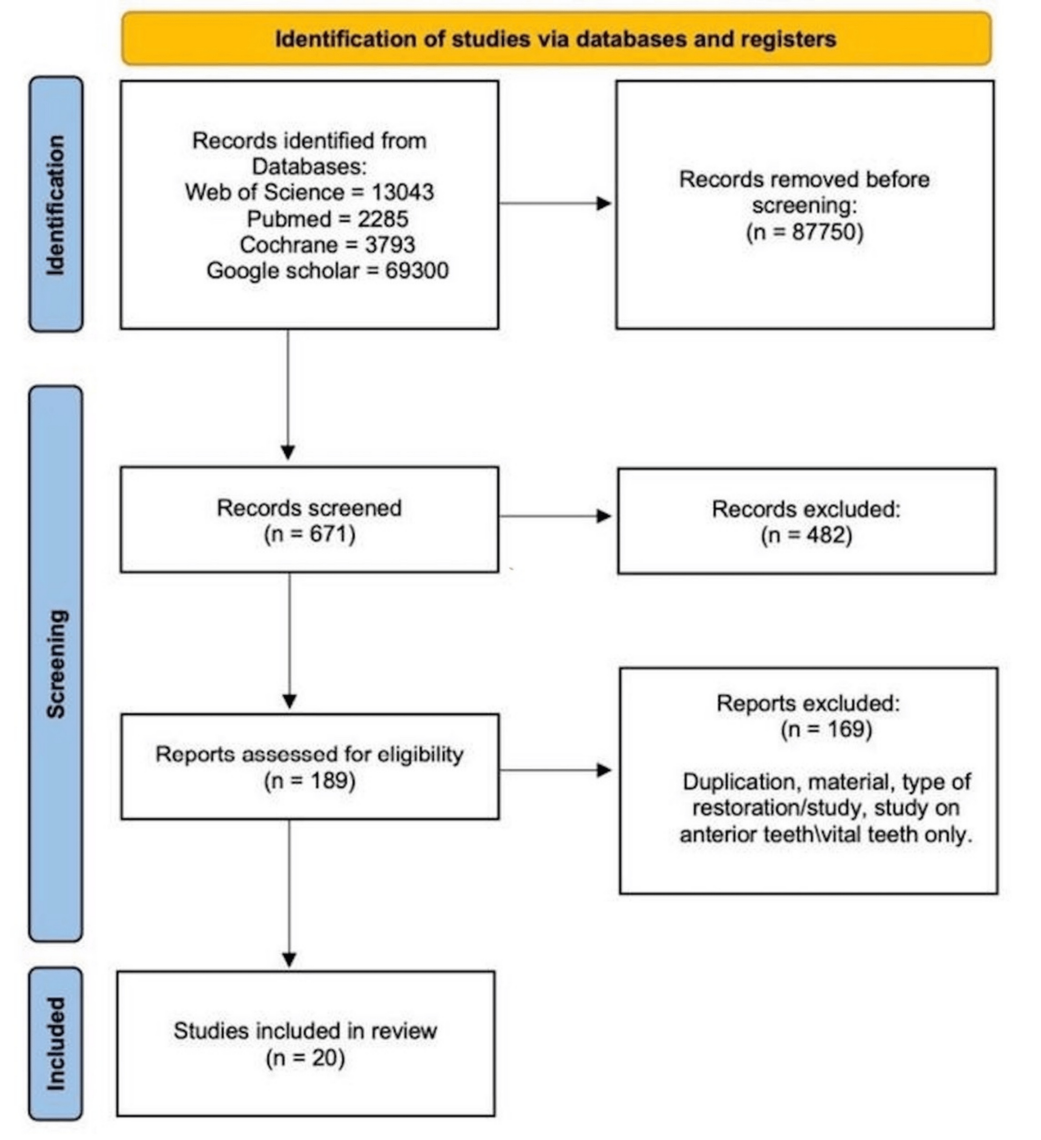
PRISMA flow diagram. PRISMA, Preferred Reporting Items for Systematic Reviews and Meta-Analysis.

A literature search through Web of Science, Cochrane, PubMed, and Google Scholar using the keywords resulted in 88,421 papers. A total of 671 articles were screened for inclusion and exclusion criteria. Four hundred and eighty-two articles that did not meet the criteria were excluded. A total of 189 full-text papers were assessed, and 20 articles were included in this study. Nineteen cohort studies and one case-control study were included and assessed in this review. According to current findings, no RCTs published met the criteria of the review. Figure 1 shows the PRISMA chart of the study selection stage [7].

Articles were categorized according to the type of studies involved, either cohort studies or case-control studies. Each type of study was assessed independently using a different type of quality assessment scale. 

The NOS was used for quality assessment of both cohort and case-control studies [10]. For cohort studies, the scale focuses on three main categories: selection, comparability, and outcome. Four maximum scores were assigned for selection: two for comparability and three for outcome. Table 1 shows the quality assessment of cohort studies using the NOS for cohort studies [10].

**Table 1:** Cohort studies quality assessment using the Newcastle-Ottawa scale for cohort studies.

Study name	Selection				Comparability	Outcome			Total score
	Representativeness of the exposed cohort	Selection of the non-exposed cohort	Ascertainment of exposure	Demonstration that outcome of interest was not present at the start of the study	Comparability of cohorts on the basis of the design or analysis	Assessment of outcome	Was follow-up long enough for outcomes to occur	Adequacy of follow-up of cohorts	
Örtorp et al. (2012) [11]	0	0	1	1	1	1	1	1	6
Kokubo et al. (2009) [12]	1	0	1	1	1	1	1	1	7
Segal (2001) [13]	1	0	1	1	0	1	1	1	6
Mikeli et al. (2022) [14]	1	0	1	1	2	1	1	1	8
Miura et al. (2021) [15]	1	0	1	1	1	1	1	1	7
Solá-Ruiz et al. (2021) [16]	1	0	1	1	1	1	1	1	7
Huettig and Gehrke (2016) [17]	1	0	1	1	2	1	1	1	8
Tartaglia et al. (2015) [18]	1	0	1	1	2	1	1	1	8
Miura et al. (2018) [19]	1	0	1	1	2	1	1	1	8
Rauch et al. (2018) [20]	1	0	1	1	2	1	1	1	8
Waldecker et al. (2022) [21]	1	0	1	1	2	1	1	1	8
Taskonak and Sertgöz (2006) [22]	0	0	0	1	2	1	1	1	6
Sorrentino et al.( 2012) [23]	1	0	0	1	1	0	1	0	4
Örtorp et al. (2009) [24]	0	0	1	1	0	1	1	0	4
Galindo et al. (2011)[25]	0	0	1	1	1	0	1	0	4
Murgueitio and Bernal (2012) [26]	1	0	1	1	1	1	0	1	6
Van den Breemer et al. (2021)[27]	1	0	1	1	2	1	1	1	8
Beier et al. (2012) [28]	1	1	1	1	2	1	1	1	9
Stoll et al. (2007) [29]	1	0	1	1	2	1	1	0	7

For case-control studies, the scale is categorized into selection, comparability, and exposure. The maximum score is 4 for selection, 2 for comparability, and 3 for exposure. Table 2 shows a case-control study quality assessment using the NOS for case-control studies [10].

**Table 2:** Case-control study quality assessment using the Newcastle-Ottawa scale for case-control studies.

Study name	Selection				Comparability	Exposure			Total score
	Is the case definition adequate?	Representativeness of the cases	Selection of controls	Definition of controls	Comparability of cases and controls on the basis of the design or analysis	Ascertainment of exposure	Same method of ascertainment for cases and controls	Non-response rate	
Mandal et al. (2022)[30]	0	1	0	0	0	1	1	0	3

Table 3 shows a comparative analysis of the 20 studies that are included in the present review. Studies were compared based on the study design, quality assessment score, material, type of restoration full/partial, follow-up duration, number of teeth, and survival rate. 

**Table 3:** Comparative analysis of included studies.

Study name	Study design	Quality assessment score	Material	Full/Partial coverage	Follow-up duration	Number of teeth	Survival rate (%)
Örtorp et al. (2012) [11]	Retrospective cohort	6	Porcelain-veneered zirconia	Full coverage	5 years	143	88.8
Kokubo et al. (2009) [12]	Prospective cohort	7	Procera AllCeram	Full coverage	5 years	75	90.2
Segal (2001) [13]	Retrospective cohort	6	In-Ceram crown	Full coverage	6 years	369	99.2
Mikeli et al. (2022) [14]	Prospective cohort	8	Monolithic zirconia	Full coverage	3 years	22	Not reported
Miura et al. (2021) [15]	Prospective cohort	7	Monolithic zirconia	Full coverage	3.5 years	40	92.8
Solá-Ruiz et al. (2021) [16]	Prospective cohort	7	Monolithic zirconia	Full coverage	5 years	50	98
Huettig and Gehrke (2016) [17]	Prospective cohort	8	Lithium disilicate	Full coverage	Up to 5-years	151	96.8
Tartaglia et al. (2015) [18]	Prospective cohort	8	Zirconia	Full coverage	7 years	130	94.7
Miura et al. (2018) [19]	Retrospective cohort	8	Zirconia	Full coverage	Up to 12-years	74	67.2
Rauch et al. (2018) [20]	Prospective cohort	8	Lithium disilicate	Full coverage	10 years	26	83.5
Waldecker et al. (2022) [21]	Prospective cohort	8	Monolithic and partially veneered zirconia	Full coverage	5 years	158	Up to 100
Taskonak and Sertgöz (2006) [22]	Prospective cohort	6	Lithium disilicate	Full coverage	2 years	<20	Not reported
Sorrentino et al. (2012) [23]	Retrospective cohort	4	Procera AllCeram	Full coverage	6 years	61	95.2
Örtorp et al. (2009) [24]	Retrospective cohort	4	Porcelain-veneered zirconia	Full coverage	3 years	168	92.7
Galindo et al. (2011) [25]	Prospective cohort	4	Alumina	Full coverage	Up to 10 years	86	84
Murgueitio and Bernal (2012) [26]	Prospective cohort	6	Leucite‐reinforced IPS Empress	Partial coverage	3 years	210	97.1
Van den Breemer et al. (2021) [27]	Prospective cohort	8	Glass-ceramic	Partial coverage	Up to 5 years	765	99.6
Beier et al. (2012) [28]	Retrospective cohort	9	Glass-ceramic	Partial coverage	Up to 12 years	547	Up to 98.9
Stoll et al. (2007) [29]	Retrospective cohort	7	IPS-Empress	Partial coverage	10 years	1624	53
Mandal et al. (2022) [30]	Case control	3	All ceramic	Partial coverage	Up to 10 years	160	92.21

Full-Coverage Crowns 

After identifying and screening the articles, and based on the inclusion and exclusion criteria, there were no RCTs evaluating the clinical outcome of full-coverage crowns in the last 20 years. Nevertheless, a literature search was conducted, which yielded 15 cohort-type studies on the clinical outcome of full-coverage crowns. The majority of the studies concluded that single porcelain crowns have high survival rates, reaching up to 95%, and excellent clinical performance, with a maximum 9% failure rate [12,13,26].

In a study that evaluated the performance of zirconia single crowns over a five-year period, Anders O”rtorp et al. in 2012 found a cumulative survival rate (CSR) of 88.3%. This finding suggests that the majority of the zirconia crowns did not experience complications throughout the duration of the study [11]. However, 9% of the crowns had been identified as failures, along with a five-year study conducted by Y. Kokubo et al. in 2009, which also demonstrates a CSR of up to 90.2%. The majority of failures were attributed to fractures in the veneering porcelain and the aluminum oxide coping. Despite these issues, 98% of the crowns were rated as satisfactory based on the California Dental Association criteria [12].

In the study conducted by Miura et al. in 2018, the most frequent clinical complication observed was chipping in the veneer ceramic. This complication was limited to the posterior region. Nevertheless, the results indicated that all-ceramic single crowns placed in the anterior region exhibited favorable clinical outcomes for at least 10 years, while the success rate of crowns in the posterior area was considerably lower during the same duration [19]. In contrast, the study conducted by Rauch et al. in 2018 found no risk of chipping. The findings indicated that the 10-year survival rate for monolithic lithium disilicate crowns was 83.5%, demonstrating a high level of longevity. Additionally, these crowns were noted to provide exceptional esthetic outcomes [20].

Monolithic lithium disilicate crowns in the posterior region exhibited slightly reduced durability compared to metal-ceramic crowns, as reported by Rauch et al. in 2018 [20]. The study conducted by Waldecker et al. in 2022 found that zirconia single crowns, whether partially veneered or monolithic, have a high rate of survival in the medium term without compromising esthetic outcomes. The only reasons for failure were biological complications [21].

Numerous studies discovered that biological complications were the cause of the failure. In 2016, Fabian Huettig et al. showed in their study excellent clinical performance, esthetics, and biocompatibility for lithium disilicate single-tooth crowns that are heat-pressed and adhesively bonded. Additionally, they provided comparable marginal integrity for all ceramic crowns. However, the performance was compromised by early events, which were heavily related to clinical handling and biological impairment [17]. Moreover, according to a study conducted by Martha L. Galindo et al. in 2011, the failures observed were mostly attributed to biological reasons rather than technical issues. The study concluded that alumina single crowns exhibit a high long-term survival rate, comparable to that of metal-ceramic crowns, with a 10-year survival rate of 95% [25].

Barry S. Segal et al. showed that high success rates for all-ceramic crowns were achieved by consistently adhering to the preparation and cementation protocols. Moreover, all-ceramic crowns with alumina cores were suitable alternatives for anterior and posterior locations and had success rates that were equal to or exceeded those of ceramo-metal crowns [13].

Several research studies demonstrate the high survival rate and good clinical performance of ceramic crowns. Aikaterini Mikeli et al. in 2022 conducted research with the objective of assessing the clinical performance of posterior monolithic zirconia single crowns that were completed in three years. They found excellent medium-term clinical performance in zirconia crowns [14]. Furthermore, a 3.5-year clinical case study on the failure analysis of monolithic zirconia crowns was carried out by Shoko Miura et al. (2021). Their results suggest that the molar application of monolithic zirconia crowns requires detailed attention to interocclusal clearance and whether the antagonist tooth has been partially restored. As the success and survival rates were as high as 90%, monolithic zirconia crowns could be an effective fixed dental prosthetic treatment option for restoration in the molar region [15].

Moreover, Ma Fernanda Solá-Ruiz et al. in 2021 determined that monolithic zirconia crowns on posterior teeth were a highly predictable treatment option with a high survival rate after evaluating the clinical behavior and survival rate at a five-year follow-up [16]. Also, Roberto Sorrentino et al. (2012) conducted a six-year retrospective study evaluating 209 all-ceramic single crowns cemented on natural and implant-supported abutments with different luting agents clinically. The crowns had remarkable outcomes, with a success rate of 90.9% and a CSR of 95.2%. These rates show how well the AllCeram crowns function in clinical settings [23].

Anders Ortorp et al. conducted a three-year retrospective and clinical follow-up study on zirconia single crowns placed in private practice in 2009. The findings indicate that zirconia crowns exhibit good clinical performance with low failure rates, making them a viable metal-free restoration option, particularly for premolars and molars. Patients expressed high satisfaction with their zirconia crowns [24].

The findings of the Gianluca M. Tartaglia et al. study showed that zirconia core crowns remain a viable clinical option for both single- and multiple-unit prostheses and have favorable functional properties even after seven years of use [18]. In addition, Burak Taskonaka et al. in 2006 conducted a clinical study that aimed to assess the clinical performance of fixed partial dentures and crowns made of lithia-disilicate. Their findings demonstrated that during a two-year period, single-unit all-ceramic crowns exhibited satisfactory clinical performance [22].

Partial Indirect BPR

Partial indirect BPRs are usually used on vital teeth, as many research protocols focus on their clinical performance on vital teeth. However, during the search across various databases, there were insufficient studies on their application to non-vital teeth. Following the identification and screening of articles, the literature search conducted based on predefined inclusion and exclusion criteria resulted in the inclusion of four cohort studies and one case-control study evaluating the clinical outcomes of partial indirect BPRs.

In 2021, Van den Breemer et al. conducted a clinical study to evaluate the clinical performance of partial glass-ceramic posterior restorations. They found excellent estimated cumulative survival and success rates after five years being 99.6% and 98.6%, respectively. The study also found that all the partial-ceramic posterior restorations were not significantly affected by tooth location, pre-restorative endodontic status, or extension of the indirect ceramic restoration [27].

However, in the findings of Stephanie Beier and his colleagues, study restorations on premolars survived longer compared to restorations on molars in the first 15 years. Thus, partial-ceramic restorations were affected by the tooth location. They were also evaluating the all-ceramic inlay and onlay restorations in both vital and non-vital posterior teeth, and there was no statistically significant difference between the two groups (p = 0.913) [28]. On the other hand, Rafael Murgueitio et al. in 2012 analyzed the survival rate and failure rate of IPS leucite-reinforced ceramic onlays and partial veneer crowns with a three-year follow-up. They reported a failure rate of up to 3.33%. Specifically, they concluded that vital teeth had a lower likelihood of failure compared to non-vital teeth, and second molars exhibited a five-fold higher susceptibility to failure compared to first molars [26]. This significant difference between the vital and non-vital teeth was also found in a study conducted by R. Stoll et al. in 2007. The results demonstrated highly significant differences (p < 0.0001) in a log-rank test. Despite this finding, after a 10-year observation period, promising results for inlays were seen, with a survival probability ranging from 80% up to 95% [29]. Similarly, the high survival rate, very low failure rate (7.6%), and 92.21% success rate of ceramic inlays and onlays that were fabricated on posterior teeth were found as a result of the Bhushan Mandal study in 2022. They also found a significant variation in survival rate between vital and nonvital teeth and between molars and premolars [30].

Beier et al. in 2012 found that the efficacy of cast gold restorations compared to glass-ceramic onlays and inlays was superior, despite the fact that glass ceramics remain successful in posterior teeth. Further, the estimated survival rates for onlays and inlays ranged between 89.6% and 98.9% after 5, 10, and 12 years of follow-up. Moreover, they considered the occlusal forces and type of occlusion by providing acrylic resin occlusal guards for bruxers to reduce occlusal forces during jaw movement, and they strictly obtained a canine-guided type of occlusion for all patients in this study. All these considerations led to a reduced risk of failure [28].

Discussion

The purpose of this review is to compare the clinical outcomes of partial indirect BPRs with full-coverage crowns for endodontically treated teeth in posterior areas. In all restorative cases, selecting the appropriate treatment option requires wise weighing. Such a choice must consider the factors that may influence the outcome of treatment.

To ensure a long survival rate, collaboration with the laboratory technician, the selection of appropriate ceramic materials, and following tooth preparation protocols with careful treatment planning are essential considerations [13]. Furthermore, rapid advances in material technology, as well as in adhesive dentistry, have led to new treatment options that are reflected in an extended range of indications and in less invasive tooth preparation designs. 

All-ceramic systems are suitable for a wide range of indications, covering almost all areas of fixed restorative dentistry, from single-unit crowns to full-mouth rehabilitation.

Several studies have been concerned with assessing the clinical outcome of full-coverage crowns in endodontically treated teeth. Most studies concluded that single crowns have high survival rates, reaching up to 95%, and excellent clinical performance [12,13,23,24].

The full-coverage crown is typically used to esthetically and functionally restore a tooth, and it can preserve the tooth from fractures following endodontic treatment. However, biological impairments like root-canal treatment typically have an adverse effect on the crown's clinical performance when compared to vital teeth [17,21,25]. Furthermore, tooth vitality can stop further tooth damage by alerting the patient and inducing pain as a preventive measure.

Restoring non-vital posterior teeth with a destroyed tooth structure could be challenging; a full-coverage crown used to be the first treatment choice for a long time. However, it is well known that conventional crown preparation techniques lead to a significant loss of tooth structure. The introduction of adhesive procedures pointed out the need to move to "prevention of extension" and made it possible to res the non-vital teeth using a minimally invasive restorative approach. Partial indirect BPRs provide cuspal coverage even though they are considered a minimally invasive alternative as they preserve more sound tooth structure. 

To the best of the authors' knowledge, no studies have summarized the available clinical data on the actual clinical performance and outcomes of indirect BPR compared to a full-coverage crown. 

Many studies have shown high survival and success rates for partial indirect BPRs. For example, Van den Breemer et al. (2021) reported excellent estimated cumulative survival and success rates after five years [27], as did Mandal et al. (2022), who found a high survival rate and low failure rate for ceramic inlays and onlays [30]. These findings suggest that these restorations can be successful over the medium to long term for both vital and non-vital teeth. The studies also suggest that several factors may affect the clinical outcomes of partial indirect BPRs, including the type of restoration (e.g., inlays vs. onlays), the material used, the location of the tooth (e.g., premolars vs. molars), and whether the tooth is vital or non-vital. Understanding these factors can help clinicians make informed decisions when choosing restorative options [16,17,19,26].

Some studies, such as those by Beier et al., compared the efficacy of glass-ceramic onlays and inlays with that of cast gold restorations. While the glass-ceramic restorations were found to be successful, their efficacy was considered inferior to that of cast gold restorations at the time of the study [28]. This suggests that the choice of restorative material should be based on factors such as longevity and clinical performance.

Thus, partial indirect BPRs have shown promising clinical outcomes, especially in vital teeth; more research is needed to fully understand their performance in non-vital teeth and how various factors can affect their longevity and success rates.

*Limitations and Recommendations* 

According to the authors’ search and based on the stated criteria, no RCTs have yet been published to meet the criteria of this review. Therefore, the study was subjected to limitations, particularly the absence of RCTs. Moreover, the studies that were included in this review assessing BPR suggest that partial indirect BPRs are more commonly used on vital teeth, with limited research available on their performance on non-vital teeth, which highlights a gap in the current literature. Thus, this study suggests the need for additional RCTs that focus on endodontically treated teeth restored with partial indirect BPRs, in addition to full-coverage crown updated articles.

## Conclusions

This study concludes that both full-coverage crowns and partial indirect BPRs demonstrate comparable survival rates, both at 95%. However, limited articles exist regarding partial indirect BPRs on non-vital teeth, indicating the need for further updated studies. Decision-making processes between both options, either a full-coverage crown or a partial indirect BPR, need careful consideration of multiple factors, such as remaining tooth structure, tooth location, occlusion, and patient oral care and hygiene. Partial indirect BPR, which provides occlusal coverage, facilitated by advancements in adhesive dentistry, holds promise as a treatment option for posterior endodontically treated teeth and necessitates its inclusion in treatment planning as an option.
